# Retraction Note: Investigation of the competitive nature of eMBB and mMTC 5G services in conditions of limited communication resource

**DOI:** 10.1038/s41598-025-98710-9

**Published:** 2025-04-28

**Authors:** Viacheslav Kovtun, Krzysztof Grochla

**Affiliations:** 1 Vinnytsia National Technical University, Vinnytsia, 21000 Ukraine; 2 Institute of Theoretical and Applied Informatics, Polish Academy of Sciences, 44-100, Gliwice, Poland

Retraction of: *Scientific Reports* 10.1038/s41598-022-20135-5, published online 26 September 2022

The Editors have retracted this Article.

Following publication, the inclusion of several questioned references in the Article was brought to the attention of the Editors. Investigation by the Editors have uncovered further issues, including irrelevant citations and significant textual overlaps with previously-published work with no common authors^1^. The Editors no longer have confidence in the claims of the Article. The Authors agree with this retraction.
